# The use of ibrutinib for the management of paraneoplastic bullous pemphigoid associated with monoclonal B-lymphocytosis: A case report and brief literature review

**DOI:** 10.1016/j.jdcr.2024.08.046

**Published:** 2024-09-28

**Authors:** Vy X. Pham, Aleksandr Itkin, Marin F. Xavier, Erik Gilbertson

**Affiliations:** a Dermatology Department, University of California, San Diego School of Medicine, San Diego, California; b Dermatology Department, Scripps Health, San Diego, California; c Hematology/Oncology Department, Scripps Health, San Diego, California

**Keywords:** autoimmune, bullous pemphigoid, chronic lymphocytic leukemia, CLL, ibrutinib, lymphoproliferative, MBL, monoclonal B-lymphocytosis, paraneoplastic, pemphigus

## Introduction

Bullous pemphigoid (BP) is a rare autoimmune disease that may often arise in association with underlying malignancies. The first presenting signs are predominantly cutaneous lesions which present as blistering eruptions and fail to respond to antimicrobial medication. Diagnosis is made using direct immunofluorescence (DIF), indirect immunofluorescence (IIF) and serological studies which indicate the presence of antibodies against basement membrane proteins. Due to the complexity and variability of histopathologic, clinical, and pathogenic presentations of paraneoplastic BP (PBP), treatment of this disease is often highly individualized. Here, we describe a case of PBP in a 73 year old male associated with monoclonal B-lymphocytosis (MBL), treated with ibrutinib, a Bruton’s tyrosine kinase (BTK) inhibitor.

## Case report

**Fig 1:**
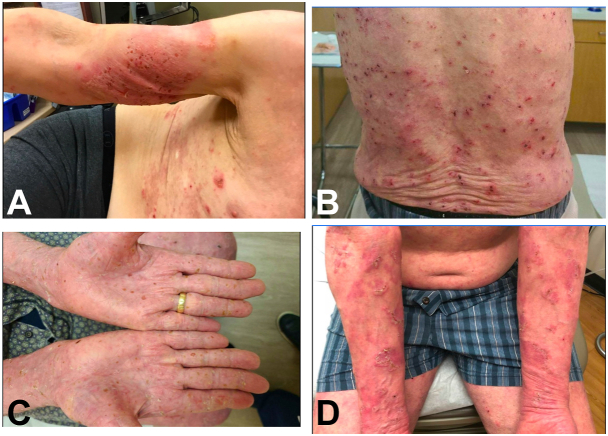
Lesions before ibrutinib. These series of clinical images are a chronological (2019 (**A**), 2020 (**B**), 2021 (**C**), 2022 (**D**)) documentation of the persistent, diffuse bullous lesions of the trunk and extremities, resistant to the multiple trialed therapies.

**Fig 2:**
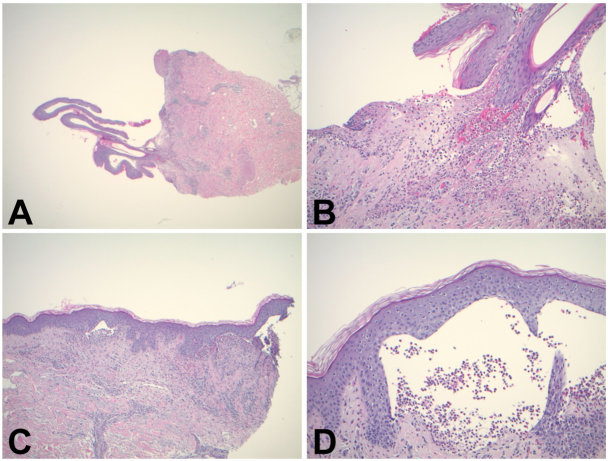
Biopsies stained with hematoxylin and eosin (H&E) stain from 2019 (**A** and **B**) at 20× and 100× magnification, respectively and 2020 (**C** and **D**) at 40× and 100× magnification, respectively, demonstrating acantholysis of the skin, resulting in blister formation.

A 73 year-old Caucasian male presented with a 1-month history of progressively worsening, diffuse bullous and pruritic plaque eruption that failed to respond to prednisone, doxycycline, and niacinamide. On physical examination, there were multiple crusted erythematous plaques with scale on the arms, chest, back, and legs, and scattered vesicles on the volar forearms (Fig 1). Initial swab testing showed no pathogenic organisms, and punch biopsy showed mixed leukocytosis with limited spongiosis. Subsequent biopsies demonstrated chronic inflammation of the dermis with eosinophilic infiltrate, acantholysis, and near complete epidermal denudation (Fig 2). DIF revealed linear C3 deposition at the intact basement membrane and IIF showed autoantibodies directed at BP antigens and type VII collagen found in the dermoepidermal junction, favoring a diagnosis of bullous pemphigoid (BP). An alternate diagnosis of cicatricial pemphigoid, epidermolysis bullosa acquisita, and paraneoplastic pemphigus was considered, but the significant eosinophilia in biopsy specimens and clinical picture of severe pruritus without intractable stomatitis argued for BP as the diagnosis.

Initial treatment with hydroxyzine, clobetasol, and mycophenolate mofetil, with the later addition of rituximab injection, showed limited improvement of pruritus and blistering. Given the lack of response, mycophenolate mofetil was substituted for azathioprine and the patient was also started on subcutaneous dupilumab injections due to highly elevated IgE levels (729 kU/L). While the IgE levels decreased (540 kU/L) after 6 months of treatment, the patient’s pruritus and recurrent vesicular eruptions persisted.

While on dupilumab, additional therapies were trialed, such as IVIG, the substitution of azathioprine for dapsone, and the addition of methotrexate injections. With all of these treatments, the patient reported temporary decreases in skin eruptions initially but would return to the clinic with recurrence of his symptoms shortly after. The patient was maintained on combination therapy of dapsone and dupilumab for approximately 1 year. During this period, the patient was found to have slightly elevated tryptase levels, and a KIT D816 V mutation was discovered by polymerase chain reaction. However, due to very minimally elevated levels of tryptase, KIT inhibitors were considered to have no role in treatment. Upadacitinib, a second-generation Janus Kinase inhibitor selective for Janus Kinase1, was briefly trialed in place of dupilumab, but yielded little improvement, ultimately being discontinued.

After discontinuing upadacitinib, a hematology work-up incidentally discovered a subclinical monoclonal population of B lymphocytes with chronic lymphocytic leukemia (CLL) morphology. A bone marrow biopsy performed showed normocellularity with no morphologic abnormalities and orderly trilineage hematopoiesis. The biopsy also showed incidental findings of kappa light chain restricted monoclonal B lymphocytes. Flow cytometry studies of the monoclonal cell population showed kappa light chain, CD5, CD11c, CD19, CD20, CD22, CD23, CD43, CD45, CD52, and CD200 expression. In the absence of lymphadenopathy and/or splenomegaly, a diagnosis of low count MBL was favored over small lymphocytic lymphoma.

**Fig 3:**
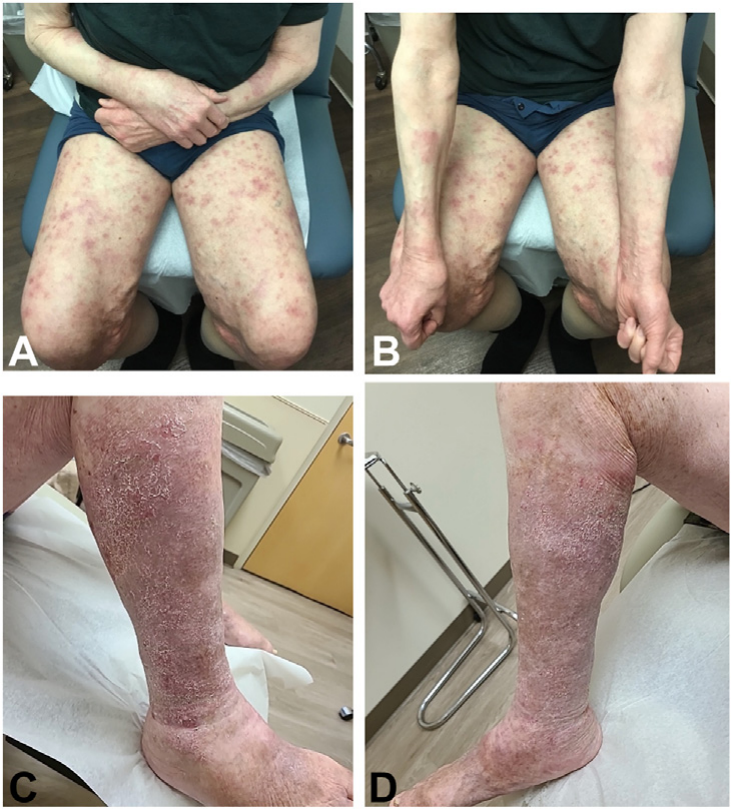
Lesions after ibrutinib. The clinical images in panels (**A**) and (**B**) demonstrate drastically reduced blister formation after 1 month of high dose ibrutinib. The clinical images in panels (**C**) and (**D**) are from a follow-up appointment after the patient developed venous stasis dermatitis 3 months after starting ibrutinib and a few weeks after being restarted on a reduced dose, demonstrating patchy scaling, but no blister formation.

Six months after the bone marrow biopsy, the patient was started on ibrutinib, a BTK inhibitor, in addition to his ongoing treatment with dupilumab and dapsone with drastic improvement in his blistering upon follow-up 4 weeks after (Fig 3, *A* and *B*). With a dose of 420 mg daily, the patient did not see any new blistering over the month, the longest period to date since his diagnosis in 2019. Of note, his most recent immunofluorescent studies in January 2024 showed negative basement membrane IgG.

Due to impetiginized stasis dermatitis and swelling of the lower extremities 2 months after initiating his new treatment, ibrutinib was temporarily discontinued resulting in a flare-up of his PBP with bullae on the trunk and extremities. After stasis dermatitis improved with medicated compression dressings, ibrutinib was restarted at 140 mg daily with bullous lesions going into remission (Fig 3, *C* and *D*). On this lowered dose of ibrutinib, the patient has been in remission for 6 months and will be monitored for any recurrence of other adverse events.

## Discussion

Low count MBL is a common diagnosis among elderly patients, occurring in about 5% of patients between 60 and 80 years old, but very few progress to CLL and most are untreated.^1^ However, given our patient’s symptomatic presentation, management with BTK inhibitors was prompted. The efficacy of BTK inhibitors is supported by a review by Ghane et al which highlights the possible role of BTK signaling in autoimmune blistering disease pathogenesis. Given the crucial role of BTK in the proliferation of B cells and downstream activation of cytokines, this enzyme has been shown to play a role in the development and survival of autoantibodies.^2^ By mitigating BTK signaling with drugs such as ibrutinib, a therapeutic effect is proposed to be achieved through this immunosuppressive mechanism.

A brief review of the literature shows a well-documented association between lymphoproliferative diseases, such as CLL, with autoantibody-driven blistering diseases, such as paraneoplastic bullous pemphigoid and paraneoplastic pemphigus (Table I). However, few have reported the presence of these diseases in association with MBL, highlighting our patient’s unusual presentation. While our review shows the use of BTK inhibitors in the management of autoimmune bullous diseases associated with advanced lymphoproliferative malignancies, such as CLL, this case demonstrates its efficacy in the management of early lymphoproliferation.

**Table I:** A brief literature review of MEDLINE and EMBASE of case reports/series describing the use of ibrutinib in cases of paraneoplastic pemphigus with CLL, SLL, or MBL

Reference	Age (gender)	Associated malignancy	Histopathologic findings	Treatment	Outcome
Chen (2023)^3^	36 (M)	CLL	C3 deposition, anti-DSG-3	(1) Methylprednisolone, IVIG, fludarabine, cyclophosphamide, rituximab (2) Plasma exchange (3) Rituximab, etoposide, vindesine epirubicin, cyclophosphamide, dexamethasone (4) Ibrutinib	No response to treatments 1 and 2. Treatment 3 resulted in complete remission, but was discontinued due to financial concerns, resulting in recurrent ulceration. Disease free with initiation of and maintenance with treatment 4 (ibrutinib).
Freund (2022)^4^	70 (F)	CLL	C3 and IgG/A/M deposition, anti-DSG-3	(1) Prednisolone, cyclophosphamide, rituximab (2) Ibrutinib (3) IVIG, dexamethasone (4) Obinutuzumab	No response or minimal improvement with treatments 1 and 2. Treatment 3 resulted in partially improved mucositis. Complete resolution of mucosal erosions with treatment 4 (obinutuzumab).
Onukogu (2020)^5^	79 (F)	CLL	IgG and C3 deposition	(1) Prednisone-- > transient resolution (2) Ibrutinib and rituximab	Treatment 1 resulted in transiet resolution with later recurrence. Treatment 2 resulted in partial resolution of the lesions.
Ito (2018)^6^	62 (M)	CLL/SLL	IgG and C3 deposition, envoplakin and periplakin antibodies, anti-DSG-3	Prednisolone, ibrutinib, rituximab	Resolution of blistering eruptions (with prednisolone and ibrutinib) and stomatitis (after rituximab added).
Lee (2017)^7^	51 (M)	CLL	IgG and C3 deposition, anti-DSG-3	(1) Rituximab, methylprednisolone (2) Ciclosporin, plasmapheresis, prednisone, mycophenolate mofetil, ibrutinib	Treatment 1 resulted in in limited improvement Treatment 2 resulted in complete remission.

*CLL*, Chronic lymphocytic leukemia; *MBL*, monoclonal B-lymphocytosis; *SLL*, small lymphocytic lymphoma.

Although a differential diagnosis of subclinical CLL is still being considered as we monitor the patient, the presence of normocytic anemia without other significant cytopenias makes a case for the diagnosis of paraneoplastic pemphigoid secondary to monoclonal B lymphocytosis.

## Conflicts of interest

Dr Xavier has consulted, advised, and served on the speaker’s bureau for Imbruvica (Ibrutinib) for which she received consultant fees and honoraria, but not in the last 12 months. Author Pham and Drs Itkin and Gilbertson have no conflict of interests to disclose.

## References

[1] Zhao L., Wang Q., Liang G. (2023). Evaluation of dupilumab in patients with bullous pemphigoid. JAMA Dermatol.

[2] Rawstron A.C., Bennett F.L., O'Connor S.J. (2008). Monoclonal B-cell lymphocytosis and chronic lymphocytic leukemia. N Engl J Med.

[3] Chen C., Xu Y., Yu J., Qian S., Xie Y. (2023). A first case of successful using of ibrutinib in treating paraneoplastic pemphigus related bronchiolitis obliterans concurrent with CLL. Front Med (Lausanne).

[4] Freund J., Trautinger F., Kopetzky G., Prillinger K. (2022). Obinutuzumab in a patient with chronic lymphocytic leukemia-associated paraneoplastic pemphigus. JAAD Case Rep.

[5] Onukogu I., Ramachandran P., Narh J., Sahni S., Joseph G. (2020). Paraneoplastic pemphigus: an indication for treatment in chronic lymphocytic leukemia. Cureus.

[6] Ito Y., Makita S., Maeshima A.M. (2018). Paraneoplastic pemphigus associated with B-cell chronic lymphocytic leukemia treated with ibrutinib and rituximab. Intern Med.

[7] Lee A., Sandhu S., Imlay-Gillespie L., Mulligan S., Shumack S. (2017). Successful use of Bruton's kinase inhibitor, ibrutinib, to control paraneoplastic pemphigus in a patient with paraneoplastic autoimmune multiorgan syndrome and chronic lymphocytic leukaemia. Australas J Dermatol.

